# Lung cancer symptom appraisal among people with chronic obstructive pulmonary disease: A qualitative interview study

**DOI:** 10.1002/pon.5005

**Published:** 2019-02-12

**Authors:** Yvonne Cunningham, Sally Wyke, Kevin G. Blyth, Douglas Rigg, Sara Macdonald, Una Macleod, Stephen Harrow, Kathryn A. Robb, Katriina L. Whitaker

**Affiliations:** ^1^ Institute of Health and Wellbeing University of Glasgow Glasgow UK; ^2^ Pleural Disease Unit Queen Elizabeth University Hospital Glasgow UK; ^3^ Institute of Infection, Immunity & Inflammation University of Glasgow Glasgow UK; ^4^ Keppoch Medical Practice Possilpark Health & Care Centre Glasgow UK; ^5^ Faculty of Health Sciences Hull York Medical School Hull UK; ^6^ PET/CT Centre Beatson West of Scotland Cancer Centre Glasgow UK; ^7^ School of Health Sciences University of Surrey Guildford UK

**Keywords:** cancer, COPD, early diagnosis, oncology, symptom appraisal

## Abstract

**Objective:**

The incidence of lung cancer is four times higher in people with chronic obstructive pulmonary disease (COPD) compared with the general population. Promotion of a shorter time from symptom onset to presentation is one potential strategy for earlier lung cancer diagnosis, but distinguishing respiratory symptoms can be difficult. We investigated how the experience of COPD influences symptom appraisal and help seeking for potential lung cancer symptoms.

**Methods:**

We conducted qualitative interviews with men (n = 17) and women (n = 23) aged 40 to 83 years with COPD. Topic guides drew on the integrated symptom‐response framework and covered symptom experience, interpretation, action, recognition, help seeking, evaluation, and reevaluation. We used the framework method to analyse the data.

**Results:**

Participants said that they attributed chest symptoms to their COPD; no other cause was considered. Participants said that family/friends noticed changes in their symptoms and encouraged help seeking. Others felt isolated by their COPD because they could not get out, were fatigued, or were embarrassed. Participants visited health professionals frequently, but increased risk of lung cancer was not discussed.

**Conclusions:**

Our study provides insight into different levels of influence on symptom appraisal and targets for intervention. Greater awareness of increased lung cancer risk and support to act on symptom changes is essential and could be achieved through a concerted information campaign. Health professionals working with people with COPD could also optimise appointments to support symptom appraisal of potential lung cancer symptoms.

## 1 BACKGROUND

Lung cancer is one of the most common cancers in the United Kingdom, with more than 35 000 deaths each year.^1^ Most lung cancers are detected at a late stage when prognosis is poor, and only 10% of people diagnosed with lung cancer will survive for 5 years.^1^ To increase lung cancer survival, improvements in the lung cancer care pathway are needed. But first, we need to know where and how to intervene for most benefit.

People diagnosed with lung cancer often have multiple symptoms, which make it hard for patients and health care professionals to act promptly in relation to help seeking or onward referral.^2^, ^3^ The presence of chronic obstructive pulmonary disease (COPD), which has similar symptoms, makes deciding to act even more difficult.^4^, ^5^ The similarity in symptoms is of particular concern because people with COPD are four times more likely to develop lung cancer than the general population.^6^ One of the problems might be that people with COPD do not know that COPD poses an increased risk of lung cancer, which is independent from the risk posed by smoking, or that health professionals do not know it or consider it salient in consultations.^7^, ^8^

People with COPD will experience ongoing lung symptoms, and attributing any changes in symptoms or new symptoms to a cause other than their existing diagnosis of COPD may be challenging. In a previous study, a history of COPD was one of the few factors independently associated with increased time before seeking initial contact with a health care professional,^9^ and qualitative research with people experiencing symptoms suggestive of lung cancer confirmed that symptom appraisal was made difficult by lung comorbidities masking new respiratory changes.^10^, ^11^, ^12^

The common sense model^13^ describes the self‐regulation of health and illness with the goal being able to manage or regulate the perceived threat. More recently, an integrated symptom‐response framework (ISRF)^14^ has been proposed (Figure S1) that provides a cross‐disciplinary model of symptom appraisal, where responses to symptoms are considered an iterative process influenced by the self, social interaction, cultural expectations, and social structure. Emphasis is on the changeable and cyclical nature of symptom appraisal, which is particularly important for COPD, where changes in existing respiratory symptoms may be a critical cue to action.

The impact of comorbidities on cancer diagnosis more generally has received little attention, and there is a lack of theoretical underpinning to inform our understanding.^15^ Furthermore, previous work considering responses to potential symptoms of lung cancer has examined symptom appraisal post cancer diagnosis^11^ or post referral^12^ but has not attempted to capture the symptom appraisal and help‐seeking process in an everyday context of people living with COPD. We used the ISRF to explore how people with COPD appraise and respond to potential lung cancer symptoms.

## 2 METHODS

### 2.1 Design

We conducted a qualitative study involving semistructured interviews with men and women with COPD in Glasgow, Scotland, UK, between July 2016 and May 2017 (Data S1).

### 2.2 Participants and procedure

Glasgow provided an ideal setting for the study because rates of lung cancer in Scotland are among the highest in the world. We recruited people diagnosed with COPD aged 40 years or older using a specialist qualitative research company. From their database of people interested in research, we sought a sample of participants who had COPD, with approximately equal numbers of men and women and people living in areas of high and low deprivation. The research company contacted potential participants with written information about the study. If the person was interested, they arranged an interview with the researcher (Y.C.). Written consent was obtained from each participant. Ethical approval was received from the University of Glasgow, College of Medical, Veterinary & Life Sciences (200150084).

We finished interviewing participants when we reached data saturation, using a stopping criterion of three interviews after new ideas stopped emerging.^16^ In total, 17 men and 23 women were interviewed, ranging in age from 40 to 87 years (Table S1).

### 2.3 Topic guide

We developed a topic guide to explore interviewees' experience of lung symptoms over the previous 6 to 12 months (Data S2). The aim of the guide was to capture the symptom appraisal process in an everyday context. The interview began with the open question, “In the last six to twelve months, have you experienced any new or changing health symptoms?” Only four participants had not experienced any new or changing symptoms in the past 6 to 12 months, and these people answered the question by going back further to describe their experience. The interview continued with semistructured questions drawing on the ISRF,^14^ including questions on symptom experience, interpretation, and action. The draft topic guide was reviewed by all authors and discussed at a multidisciplinary advisory group meeting, including a patient representative. Following the initial batch of 10 interviews, it was striking that interviewees did not mention cancer; therefore, the topic guide was adjusted to include a fuller discussion of lung cancer at the end of the interview.

### 2.4 Analysis

Interviews were digitally recorded, transcribed verbatim, and imported into the software package NVivo, version 11 (QSR International, Melbourne, VIC, Australia). We analysed the data using the framework method,^17^ which allows organisation of data according to key themes and concepts. Following familiarisation with the transcripts by Y.C., K.A.R., and K.L.W., an initial thematic coding frame was developed and then discussed among the wider research team. The themes were based on the ISRF as per the topic guide. There was little evidence of additional themes relating to symptom response emerging. The agreed themes were applied to extract data from the transcripts for further interpretation. We arranged the framework matrix in a spreadsheet containing one data‐generated theme per worksheet with subthemes in the columns. The rows represented individual participants. Y.C. populated the framework matrix with relevant data extracts from the transcripts and summarised each theme with representative extracts. K.A.R. and K.L.W. reviewed the summaries and discussed them with Y.C. to check consistency with the data. Data requests should be addressed to the corresponding author.

## 3 RESULTS

**Figure 1:**
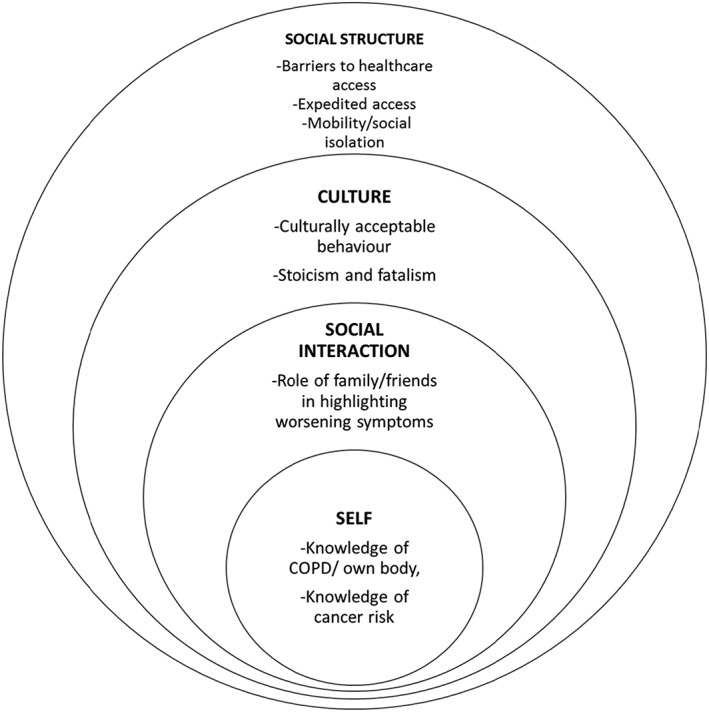
Thematic structure according to the integrated symptom‐response framework^14^

Participants were interviewed in their own homes, apart from two who requested to be interviewed in cafes. Interviews lasted an average of 42 minutes (range: 24‐72 min). The analysis was organised into four main “circles of influence” on the response to symptoms (self, social interaction, culture, and social structure; Figure 1). Each influence is described with supporting quotes from participants, along with their participant number, gender, and age.

### 3.1 Influence—self

Knowledge of COPD and their own body were prominent in participants' accounts. Participants described having always had “chest problems”; eg, they had suffered from asthma since childhood and then later developed COPD. Participants emphasised that their long history of COPD meant that they had come to know their own bodies and felt they had the expertise and confidence to advise their doctors on appropriate treatment. One participant described how she had educated herself about her condition.

I think I know all about my lungs now. I think. I am quite educated …. when I took the pneumonia there was different names used, and I wasn't sure what they meant. And, I looked that up. [P6, female, age 64]

Participants said that because they knew their body so well, they were able to make sound judgements about help seeking.

Actually, but I think your body's the best doctor, you know? Tells you everything. [P4, male, age 78]

When prompted to think of what could cause changes to their chest symptoms, participants tended to draw on external factors such as smoking, the weather, or age:

It's always worse most winters, because we are going into the damp cold weather. That's when I am worse. In the summer time it's more like, it's reverting to my asthma, you know, allergies to the pollen and things like that. [P12, male, age 52]

Participants always drew on their experience of COPD when attributing symptoms beyond external factors such as the weather or illness. It did not seem to occur to them that a change in symptoms could have a cause other than COPD.

I just thought it was down to the COPD. Yeah, I did not think it was anything else really, just, I just thought it was, I know it's a progressive illness. So I thought well it's getting a bit worse. [P14, female, age 67]

Participants were unaware that people with COPD are at increased risk of developing lung cancer and attributed worsening symptoms to their COPD. Many expressed surprise when the researcher informed them of this near the end of the interview.

No, I never gave cancer a thought I was just thinking of the breathing I never actually, … … you are saying to yourself, well, if this gets really worse, like what I am saying, you just keep putting it down to that [COPD]. [P33, female, age 61]

### 3.2 Influence—social interaction

The role of other people was central; family members could draw attention to bodily changes, such as noticing a worsening cough or changed pallor:

Or if I am getting up in the morning and going to work or something like that, if my mum's round, she'll be like, “Think you need to put a bit more blusher on,” you know? She can see it. She can see that colour draining from me. [P9, female, age 45]

Participants also provided examples of other people noticing symptoms before they did.

They would notice the wheeziness when I am sitting down. Short of breath when I am sitting down as well. That's what they would notice and they would notice the wheeziness and just probably the way I look, kind of grey. [P32, female, age 53]

Participants talked about family members encouraging them to seek help for a changing or worsening symptom. Participants described how family members would even go so far as to arrange appointments or drive them to the hospital:

They come up and they say, no, we are not waiting. Telly goes off, bag ready. “you are going to the hospital.” [P3, female, age 69]

COPD participants also talked about how the condition was socially isolating and reduced their opportunity for social interaction because of the physical limitations of the illness:

I do not have much of a social life now … …. So I have sort of socially excluded myself from a lot of things. Because I cannot keep, I cannot really keep up with any of it, you know, any walking or anything like that now, you know. [P25, male, age 68]

Participants described deliberately avoiding social situations where symptoms would become apparent because they perceived them to be embarrassing or did not want people to notice them:

I hate staying with anybody, because, the noise of my chest, and coughing on, and—maybe—we went away, crowd of women …. And it was one woman, and she went, “Oh, I could not sleep with you.” Oh, I felt awful—I cried my eyes out. [P3, female, age 69]

This resulted in participants describing that they had fewer people in their lives, which may also impact on help seeking:

I think at one time I was sociable and now there is virtually nobody in my life so. [P12, male, age 52]

### 3.3 Influence—culture

In talking about how they respond to changing or new chest symptoms, participants portrayed the role of being a “good patient”:

Yes, mm. “Persistent three weeks on with chest infection”—well, obviously I would go to the doctor's immediately. “A cough that does not …” Go to the doctor's immediately. [P1, female, age 74]

Another feature of culturally acceptable responses was to appear stoical in response to symptoms. Participants were keen to be seen to “not make a fuss” or as time‐wasters.

I do not know, it's just a thing I do. I am not one for running to the doctor, to be honest with you. [P17, male, age 58]

Participants also emphasised that poor health was something that had to be accepted.

I mean, look at—you have just got to accept that that's the way it is. … Yeah. [P1, female, age 74]

In more extreme cases, this view led to a fatalistic attitude, as it did with one participant, who was weary of “putting up with” his poor health:

I am 55, I have lived my life, I would like to see my granddaughter grow up but really I have had enough. [P12, male, age 52]

The role of smoking also influenced how participants responded to symptoms. Participants acknowledged that smoking is an increasingly culturally unacceptable behaviour, particularly for people with COPD. In some instances, the stigma associated with continued smoking made participants reluctant to seek help because they felt the doctor would blame smoking for their symptoms:

… … they'll just tell me it was the fags [cigarettes]. Do you know what I mean? [P2, male, age 60]

### 3.4 Influence—social structure

Our participants described the influence of social structure on potential responses to new or changing symptoms and help‐seeking behaviour. Participants talked about barriers to accessing care, which included scheduling appointments outside of usual working hours and difficulties in obtaining an appointment.

Well the only thing I could do is you know go and see the doctor, see what he thinks. As I say, I know they are under a lot of pressure, but I think you know, it's easier to get in and see the pope than it is to see our doctor. [P13, male, age 76]

Barriers to accessing care and health services also included the physical challenge of travelling to the health centre, which is particularly relevant for COPD patients who may have reduced mobility.

Because I cannot get a bus from here down to my doctors. I can get two buses but the problem with that is it runs every half hour and the two of them run at the same time. [P21, female, age 54]

While difficulties in accessing care were talked about among our participants, they also described that they had better access to health services because of their COPD diagnosis. Doctors acknowledged the seriousness of their symptoms and accommodated them with emergency appointments or prescriptions.

I have got what they call “save” antibiotics in the house. And that's good because that takes away some of the worry. [P36, female, age 65]

## 4 CONCLUSIONS

This study considered how the experience of COPD influences symptom appraisal and help seeking for potential lung cancer symptoms. Drawing on the ISRF,^14^ our analysis identified key influences across many levels, including self, social interaction, culture, and social structure. A common thread was that having an existing explanatory model impacted not only on how participants interpreted and responded to symptoms but also in how social interactions and structures, described within the ISRF, responded in the presence of this existing morbidity. This made it difficult for patients and health care professionals to consider the possibility of lung cancer in response to a new or changing lung symptom, despite people with COPD being at higher risk of lung cancer.

Participants in the current study did not identify any potential link between their symptoms and lung cancer, nor recognise that they were at higher risk, even after specific probing by the interviewer. This is consistent with previous research, which reported that public awareness of COPD as a lung cancer risk factor, independent of cigarette smoking, is low.^8^ COPD participants attributed new or ongoing respiratory changes to their existing condition or at most considered their symptoms to be exacerbated because of other external factors such as the weather. There was no evidence that social interactions with friends or family or health care professionals themselves discussed alternative explanations (eg, the possibility of cancer), and therefore, their existing explanatory model remained unchallenged.^18^

The ready attribution of new symptoms to innocuous explanations but not to the presence of a new, frightening illness such as lung cancer could be due to what Anderson et al^19^ called “optimistic bias,” the tendency to favour nonthreatening explanations to those that are threatening. Although participants appeared to look for innocuous explanations, friends and family could trigger help seeking in particular by highlighting visible symptoms. The importance of interpersonal relationships in help seeking has been observed before^20^, ^21^ and specifically in the context of lung cancer.^11^

Moving beyond social interaction to wider cultural influences, we identified a concern among COPD participants about being labelled a “time‐waster” and valued cultural attributes of stoicism. Worry about wasting the doctor's time is a well‐recognised barrier to prompt help seeking^11^, ^22^, ^23^ and demonstrates the complex and delicate moral balance patients have to make between responsible use of health care services and not taking unnecessary risks with their health.

Despite having a recognised chronic condition requiring medical involvement, participants were keen to distance themselves from those considered time‐wasters and instead stressed that they would consult their doctor only when absolutely necessary. Stoicism and acceptance of poor health also manifested as fatalism, with one man (aged 55 years) saying wearily “I've lived my life.” Fatalism has previously been identified as a barrier to medical help seeking,^24^ and this is important because raising awareness of a link between COPD and cancer may be detrimental if it leads to increased fatalism. Raising awareness of the link between COPD and lung cancer should therefore be conducted alongside raising awareness of the benefits of early diagnosis.

According to the ISRF,^14^ social structure can influence responses to symptoms directly (eg, through health care access) or indirectly (though social networks' access to resources, knowledge, and so on). We observed evidence for both. Firstly, there was a juxtaposition between commonly identified access barriers to help seeking, alongside expedited access due to having COPD. Different dimensions of accessing health care were highlighted, including lack of availability and problems with accessibility.^25^ Conversely, participants described expedited access or “short‐cuts” to accessing medical care because of their condition. Although this may appear helpful on one level, it may also result in health care professionals attributing new or changing symptoms to existing medical conditions rather than considering alternative explanations.

Social isolation also impacted on help seeking—COPD participants described lack of mobility and the need to hide embarrassing or worsening symptoms. Evidence of social isolation in COPD patients has been identified before,^26^ but our study highlights that this may have wider consequences on consulting behaviour.

A major strength of this study is that it is the first to examine the process of symptom appraisal of potential warning signs for cancer in patients with an existing health condition prior to the potentially biasing effects of cancer diagnosis or referral on suspicion of cancer. The study adopted a theoretically driven approach to capture the symptom appraisal process in an everyday context for a diverse sample of men and women of relatively low socioeconomic status.

### 4.1 Study limitations

Glasgow was purposively identified as a good site for the study because of high prevalence of COPD and lung cancer; however, we acknowledge that our sample is geographically limited. We used a specialist qualitative research company for recruitment that may have biased our sample because these people are interested in taking part in research. However, on the basis of informal feedback from participants, it is our belief that this form of recruitment allowed us to reach participants who would not have engaged with more conventional approaches.

### 4.2 Clinical implications

Our study provides insights into how to intervene at different levels of influence to improve the likelihood of patients and health care professionals suspecting and acting on potential lung cancer. Studies testing health care–based interventions addressing symptom awareness/normalisation and fatalism in patients at high risk of lung cancer are ongoing.^27^, ^28^ Our study highlights the potential need for similar interventions in patients with COPD, and we have developed recommendations in order to help mitigate specific issues within this sample (see Box 1). For example, raising the awareness of the link between cancer and COPD in patients will help in identifying symptoms as something different, but this needs to be dealt with sensitively to avoid increased fatalism. For those who are socially isolated, encouraging engagement with COPD services, such as pulmonary rehabilitation programmes, may provide social support, in addition to providing another opportunity to engage with symptom awareness messages.

**Box 1:** Recommendations

Finding	Recommendations
Cancer was not considered a potential explanation for chest symptoms.	•Alert people with COPD to risk of lung cancer. •Alert people with COPD to consult with changes in symptoms. •Encourage relevant charities/support groups to include information about lung cancer risk in communications.
Family or friends noticed changes in symptoms and encourage help seeking.	•Provide information aimed at empowering patients and family members to speak about symptoms and take action. •Encourage family members to attend consultations.
People felt isolated by their COPD.	•Encourage patient engagement with COPD services such as pulmonary rehabilitation programmes or other well‐being/exercise classes to gain social support from others with COPD.
Participants visited health professionals frequently, but lung cancer risk was not discussed.	•Alert health professionals to be vigilant to lung cancer risk in COPD patients. •Move away from a “disease silo” approach. Consultations could include the question from the health professional, “is there anything else?” This recommendation is transferable to other patient groups with pre‐existing conditions at risk of developing cancer.

COPD, chronic obstructive pulmonary disease.

Emerging evidence suggests that doctors may be reluctant to openly discuss cancer with their patients.^29^ Another avenue may therefore be to help doctors recognise symptoms as a presentation of possible cancer and encourage open and honest conversations between patients and doctors about the link between COPD and cancer.

The present study advances our knowledge of the process of symptom appraisal among people with an existing chronic health condition by highlighting the influence of individual, social, and cultural factors and how these may culminate in a later diagnosis of lung cancer. We make a number of recommendations to optimise lung symptom appraisal and prompt help seeking for people with COPD.

## CONFLICT OF INTEREST

None.

## Supporting information

Data S1. Supporting information

Figure S1. ‘Concentric circles of influence’ representation of the Integrated Symptom Response Framework [14]

Table S1: Participant characteristics

Data S2. Supporting information
