# Supplementary material for: Lung cancer symptom appraisal among people with chronic obstructive pulmonary disease: A qualitative interview study
Source: Psychooncology. 2019 Feb 12;28(4):718–25. doi: 10.1002/pon.5005 (PMC6492269; doi:10.1002/pon.5005)
Supplement: Supplementary file 2 — Figure S1. ‘Concentric circles of influence’ representation of the Integrated Symptom Response Framework [14] [file PON-28-718-s002.docx]

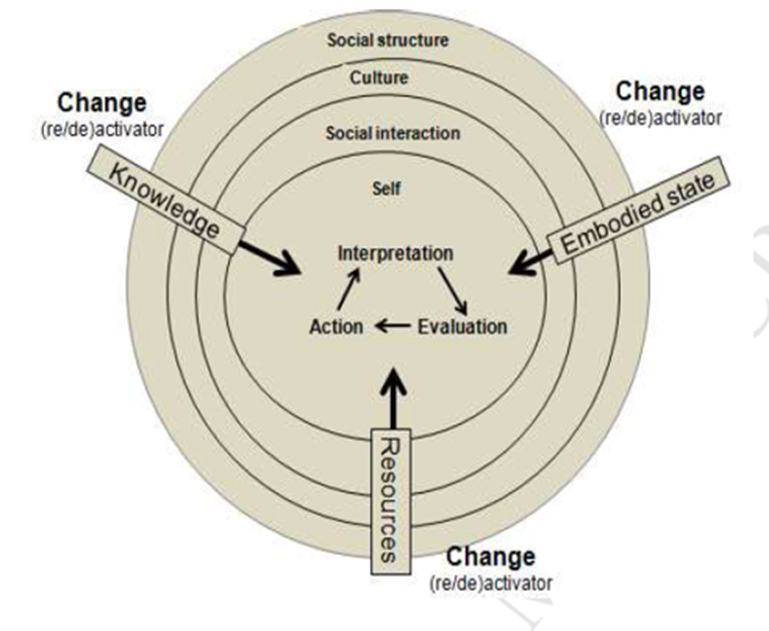


*Supplementary Figure 1*. ‘Concentric circles of influence’ representation of the Integrated Symptom Response Framework [14]
