# Supplementary material for: Lung cancer symptom appraisal among people with chronic obstructive pulmonary disease: A qualitative interview study
Source: Psychooncology. 2019 Feb 12;28(4):718–25. doi: 10.1002/pon.5005 (PMC6492269; doi:10.1002/pon.5005)
Supplement: Supplementary file 3 — Table S1: Participant characteristics [file PON-28-718-s003.docx]

Supplementary Table 1: Participant characteristics

|  | n |
| --- | --- |
| Gender |  |
| Female | 23 |
| Male | 17 |
|  |  |
| Education |  |
| School | 30 |
| Post-school training | 5 |
| University degree | 5 |
|  |  |
| Age |  |
| 40-49 | 6 |
| 50-59 | 8 |
| 60-69 | 14 |
| 70-79 | 11 |
| 80-89 | 1 |
|  |  |
| Marital status |  |
| Married/loving with partner | 24 |
| Divorced/separated | 7 |
| Widowed | 5 |
| Single/never married | 4 |
|  |  |
| Smoking status |  |
| Smoker | 7 |
| Ex-smoker^[[1]](#footnote-1)^ | 27 |
| Never smoked | 6 |
| Existing health condition |  |
| Cardiovascular | 21 |
| Musculoskeletal | 8 |
| Respiratory | 5 |
| Endocrine | 4 |
| Mental health | 6 |
| Renal | 2 |
| Haematological | 2 |
| Ear, nose, throat | 1 |
|  |  |

1. Participants reported giving up smoking for a variety of reasons. Some gave up immediately on getting their initial diagnosis of COPD, or after another serious health scare such as a heart attack. For others, it took a period of time before they gave up. [↑](#footnote-ref-1)
