# Supplementary material for: Lung cancer symptom appraisal among people with chronic obstructive pulmonary disease: A qualitative interview study
Source: Psychooncology. 2019 Feb 12;28(4):718–25. doi: 10.1002/pon.5005 (PMC6492269; doi:10.1002/pon.5005)
Supplement: Supplementary file 4 — Data S2. Supporting information [file PON-28-718-s004.docx]

Interview Topic Guide

| *Opening* |
| --- |
| 1. Personal introductions |
| 1. outline study purpose |
| 1. outline motivation for conducting study |
| ensure to mention:   - - Timing (~ 1 hour)   - Confidentiality   - Anonymity in report writing   - No right or wrong answers   - How they will know the interview has ended |

| *Body* |
| --- |
| **Topic 1: Identifying a shared vocabulary** |
| - Is there a different term for Chronic Obstructive Pulmonary Disease that you would prefer me to use during this interview?   - COPD/lung illness/lung disease/chronic bronchitis/emphysema/chronic obstructive airways disease/smokers disease |
| **Topic 2: Lung symptoms - experience & interpretation** |
| - In the last six to twelve months, have you experienced any new or changing health symptoms? *If struggling to answer prompt participant with list of symptoms from Lung Cancer Awareness Measure (Lung CAM).*    - Could start by asking them how things have been in the last year   **If yes;**   - When did you first notice changes?   - What made them notice   - What was happening at the time   - Who noticed them   - Did something about their body feel different   - At that time, what had they thought was causing it   - Have their thoughts about this changed over time   - **If so**, how - Are you still experiencing the symptom / symptoms? - How are you now when you experience one of these symptoms?   - Their thoughts – what goes through your head?   - Their emotions – how do you feel about that? - [show participant Lung CAM, if have not already done so] Are there any symptoms here, which you have not already mentioned, that you have experienced in the last 6-12 months?   **If no;**   - [establish a third person scenario with guidance from participant and continue interview through referring to this scenario] |
| **Topic 3: Lung symptoms – action** |
| - How did you react the first time you noticed you had one of these symptoms? - And what do you/would you do if you experience the symptom or if you were to experience it again? *move to next main question if GP is mentioned**   - What are their thoughts/feelings about this   - Do they see it as easy/ simple to do   - If not, why - [show participant Lung CAM] Would any of these symptoms prompt you to seek advice from a Health Care Professional   - **If yes,** which symptoms   - **If no,** why not   Before you were diagnosed with COPD what did you think was causing your symptoms? |
| **Topic 4: Symptom recognition & help seeking** |
| - Did you try to find out more about your symptom?   - Internet/ family/ friends/ health care professional   - Has this changed - Did you talk to anyone else about the symptom?   - How do they feel about telling others about their symptoms/ Do they prefer to keep it to themselves - Do you know other people who have had similar symptoms?   - Do they think it’s a common symptoms - What do your family/friends think about your symptoms?   - Do they think it’s a serious problem?   - Do they generally encourage you to seek help? Do you trust their judgments? - *Have you been to a GP/other health care professional about your symptoms?   **If yes;** and how was that?   - - What prompted them   - Did they wait some time before deciding to see GP/make appointment   - If so, what made them wait   - How many times have they seen their GP about this symptom/health complaint/concerns   - What was the first consultation like/ were their concerns dealt with/ what was the outcome of the visit   - If they have seen GP more than once, how were these visits   - Do they feel they received answers to their concerns/ have a better idea of what to do about their symptoms   - Do they try to see the same GP   - If so, why/why not?   Have you ever been to see the GP and it didn’t go well? That you weren’t happy?  I interviewed a man last week and he didn’t like the young GPs, and I had a lady the week before that said she was sick and tired of locums.  **If no;** Have you thought about seeing a GP/other health care professional?   - - What would prompt them to talk to a doctor/nurse, if anything   - Would they rather talk to someone else - If you were concerned about a symptom, what might stop you from going to the GP?   - Are there any personal reasons that would keep you from going?   - Would it affect other people if you went? Would anyone say anything to you?   - Is there any difficulty in getting there? Anything about this area that makes it harder for you to go?   - Some people say they feel a bit embarrassed. Would you be like that?   - Some people worry they are wasting the GP’s time. Would you be like that?   - Personal/ Social/ Environmental/ Financial/embarrassed/worried wasting GP time - How may these barriers be overcome?   - Again, is this something someone else could help with   - Why them/ How could they help   - Would it be made easier if something were changed in their community - Have you taken any other approaches to deal/manage with your symptom? |
| - Do you think that having COPD puts you at risk for other conditions? What about other lung conditions? |
| **Topic 5: Lung symptoms - evaluation & re-evaluation** |
| - Do you keep an eye on your symptoms?   - How do they do this   - What helps to notice changes - Would you see noticing changes in symptoms as something someone else could help with?   - Someone they know/ family/ friends/ community nurse/ health worker   - Why them/ How would they help |
| **Topic 6: Information and resources** |
| - What kind of help would you like if you noticed a symptom that you were worried about/ didn’t quite understand/ recognise? *It may help to discuss in terms of other people*   - Is there anything they think the health services do well   - Could anything be done better/anything new they could be doing   Give an example of an intervention and ask them to comment on it |

| *Closing* |
| --- |
| 1. Signal interview coming to an end 2. Is there anything else you wanted to discuss?    - In terms of noticing symptoms    - Getting help/ support/ advice    - Anything you think I have missed/ wanted to add/ think should be included 3. Give thanks for time and contribution to the research 4. Read out and complete demographics questionnaire   Amendment following first ten interviews:   - - 1. Explain study funded by Cancer Research UK because people with COPD have an increased risk of developing lung cancer.   - What do you think about this?   - Do you find this surprising? |
|  |

Thank you for taking part in this interview today. I you have no more questions, can I just take some more details from you? (Read out and get participant to complete demographics questionnaire).

We are telling everyone who takes part in the interview that if they experience persistent symptoms they should go to their GP for advice. If you have been to your GP, but the symptom hasn’t resolved, you should go back. (Explain to the participant that we are not medically qualified but can answer some questions. Refer them to NHS Choices for more information about symptoms.)

OK, now I will give you a bit more information about this study. I can also post this to you or send it by email attachment. (Refer to debriefing sheet).
